# NEURODEGENERATION BIOMARKERS HELP EXPLAIN ASSOCIATION BETWEEN INFLAMMATION AND COGNITIVE DYSFUNCTION

**DOI:** 10.1093/geroni/igad104.0329

**Published:** 2023-12-21

**Authors:** Eric T Klopak, Eileen M Crimmins

**Affiliations:** University of Southern California, Los Angeles, California, United States; University of Southern California, Los Angeles, California, United States

## Abstract

There is past evidence that immune aging (or immunosenescence) and high systemic inflammation are associated with cognitive dysfunction and ADRD. However, it is unknown whether these immune-related aging processes are associated with neurodegeneration, tauopathy, amyloid accumulation, and/or vascular issues in the brain.

To address this gap, we utilized a set of recently assayed highly innovative markers of neurodegeneration, biomarkers of inflammation, and proportions of naïve lymphocytes (indicative of less immune aging), and cognitive dysfunction (errors on the Telephone Interview for Cognitive Status) in the Health and Retirement Study, a nationally representative sample of US adults over age 50 (N = 3985).

Using structural equation modeling (SEM), we estimated latent factors representing neurodegeneration (neurofilament light chain (NfL), glial fibrillary acidic protein (GFAP), and phosphotau 181 (pTau181)), inflammation (c-reactive protein, IL10, IL1 receptor agonist, IL6, TNF receptor 1, albumin, and percentage of neutrophils), and immune aging (percentages of naïve CD8+ and CD4+ T cells). Results indicate inflammation is associated with neurodegenerative biomarkers (β = .31, p < .001) and with cognitive dysfunction (β = .08, p < .001) after controlling for age, race, gender, education, BMI, smoking, drinking, and immunosenescence. Neurodegenerative biomarkers mediated about half of the association between inflammation and cognitive dysfunction.

It appears that greater systemic inflammation may be associated with brain damage and tauopathy. Interventions focused on reducing systemic inflammation may be successful in reducing neurodegeneration and cognitive dysfunction in older adults.

